# A Blind Policy

**Published:** 1921-06-11

**Authors:** 


### THE PUBLIC WELL-BEING.

### A Blind Policy.

Last week we called attention to the controversy prevailing in this country as to the best methods of combating the great scourge of venereal disease and urged the need for common action. The announcement of a forthcoming North European Conference on Venereal Diseases would appear to suggest that public opinion throughout the civilised world is at last being roused to the importance of adopting a universal policy, which can be applied by all nations to the task of extinguishing this human plague. Even before the event takes place, however, there are indications that the fires of an undesirable controversy are being fanned to fresh heat. It is of the utmost moment that every variety of organised opinion should be represented at this Conference in order that there shall be a free, full, and candid discussion of the differing points of view, medical and moral. Yet, for some unexplained reason, only one of the British societies engaged in the anti-venereal campaign is to be represented. The National Council for Combating Venereal Diseases is to be heard, but the British delegates do not include a single member of the Society for the Prevention of Venereal Disease.

Now that is a singularly unfortunate omission, open to suspicious comment, seeing that the latter organisation, unlike the former, courageously advocates immediate self-disinfection as a means of protection—a policy which receives the support not only of the vast majority of medical men in this country, but of a large body of lay opinion. There can be few people—among medical men no less than among religious and social workers—who fail to realise the importance of the moral factor and the need for preaching self-restraint and the anti-social dangers of a promiscuous sex life. But those who pin their faith solely to this method of approach and rule out as morally improper the provision of means to prevent the consequence of infection are living in a fool's paradise and doing a serious disservice to the cause which they have at heart. If there is a movement on foot to eliminate from the Conference advocates of the movement for organised self-disinfection the Conference might as well never take place.

The chief criticism levelled against the Ministry with regard to the treatment, control, and prevention of venereal disease has been that too much money has been mere advertisement, and that too little attention is being paid to therapeutics. In the recent debate which took place in the House of Commons on the policy of the Ministry, Sir Alfred Mond, who amply demonstrated the useful activities of his Department (continued with no such sensational break as was predicted when Dr. Addison resigned), pointed out that it is necessary to proceed steadily with propaganda, as the intelligence of the public is the direction from which the real improvement in a lamentable state of affairs must be expected. The circular now issued is a kind of official compromise between two extreme views on what is admittedly a difficult and complex problem. The circular takes note of the social and moral side of the question, and, at the same time, is indirectly instructive to those who may be helped either by continuance or by prompt resort to medical aid. The Ministry declines to make an official policy of methods of self-disinfection, and, therefore, declines to issue disinfectants or to supply literature recommending their use or explaining their nature. At the same time, it advises county councils and county borough councils that leaflets should be prepared for distribution. We find it difficult to distinguish the difference between issuing such leaflets direct from the Ministry and giving instructions for them to be issued by local authorities. But Sir Alfred Mond's Department no doubt finds it politic for the time being to adopt this air of detachment, and we need not split hairs if in the result the desired purpose is achieved. Further, it should be noted that chemists are now to be allowed to supply disinfectants to those who ask for them, provided that no written or printed recommendation or instructions as to use accompany them. The weakness of this carefully qualified permission is, first, that the sanction is open to abuse because no public guidance is given as to the nature of the disinfectant, or no effective limits of choice laid down, so that more harm than good may be done in the confidence of ignorance; and, secondly, that this is only a kind of half-recognition of the fundamental fact that the prevention of disease, as *The Times* frankly states, is "necessarily and primarily a medical matter." The new sympathetic attitude of the Ministry of Health is distinctly an advance upon its former point of view, and, if it does nothing else, it may do something towards healing the regrettable dissensions between the Society for the Prevention of Venereal Disease and the National Council for the Control of Venereal Disease.

